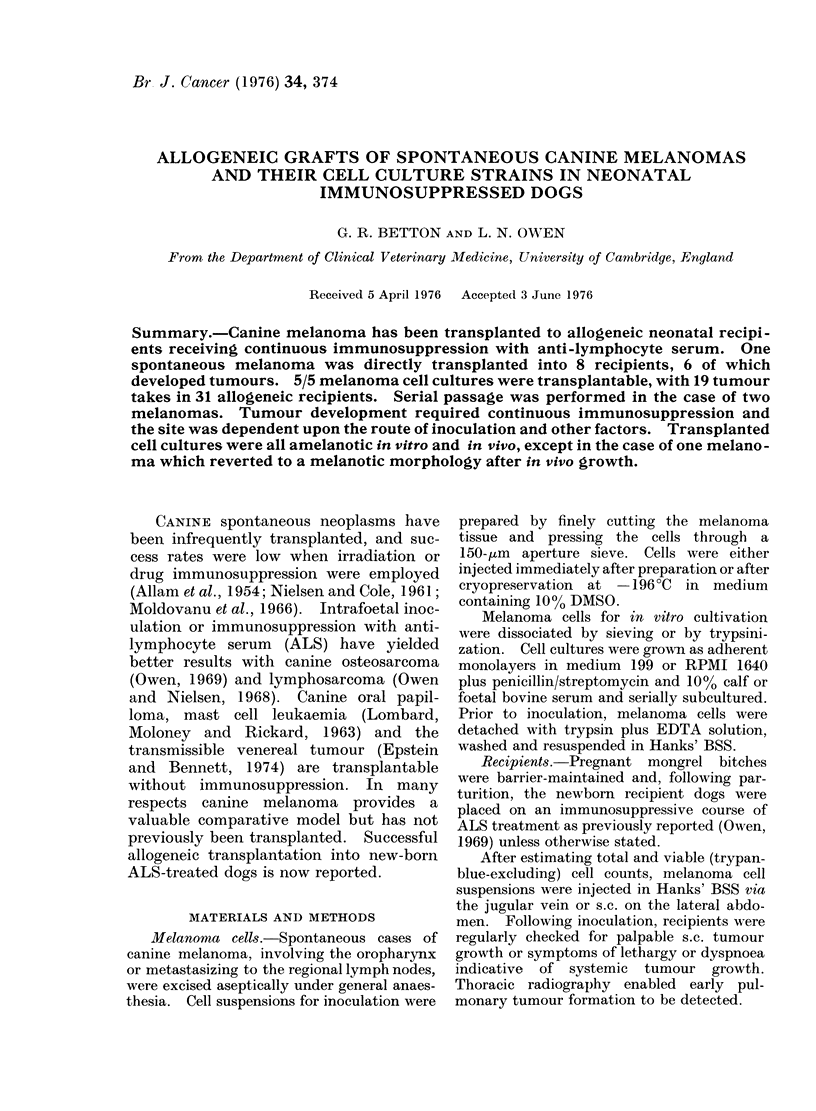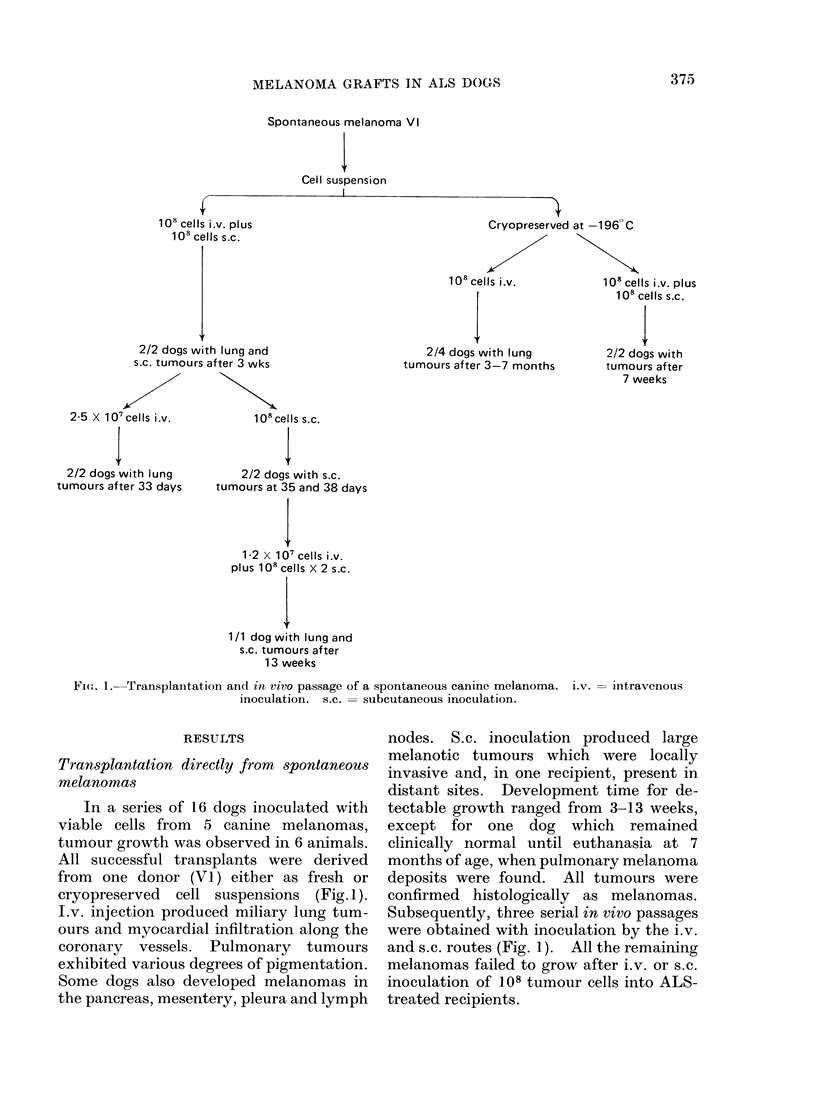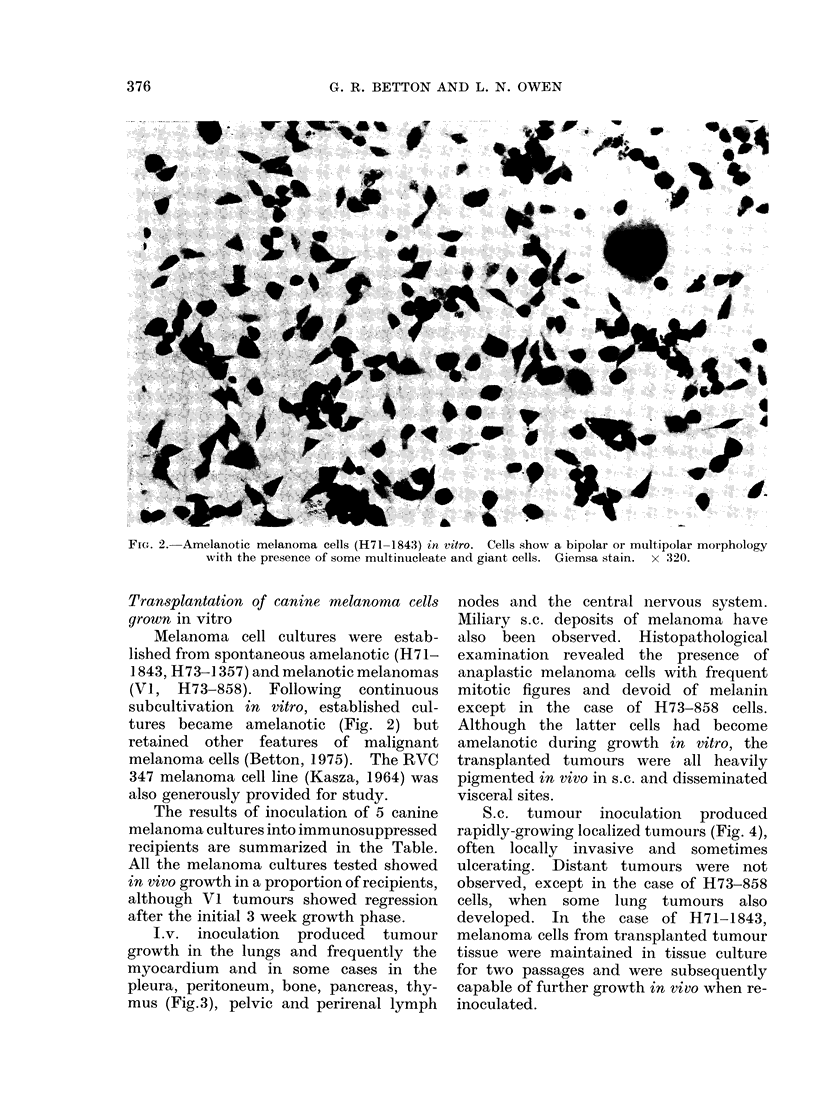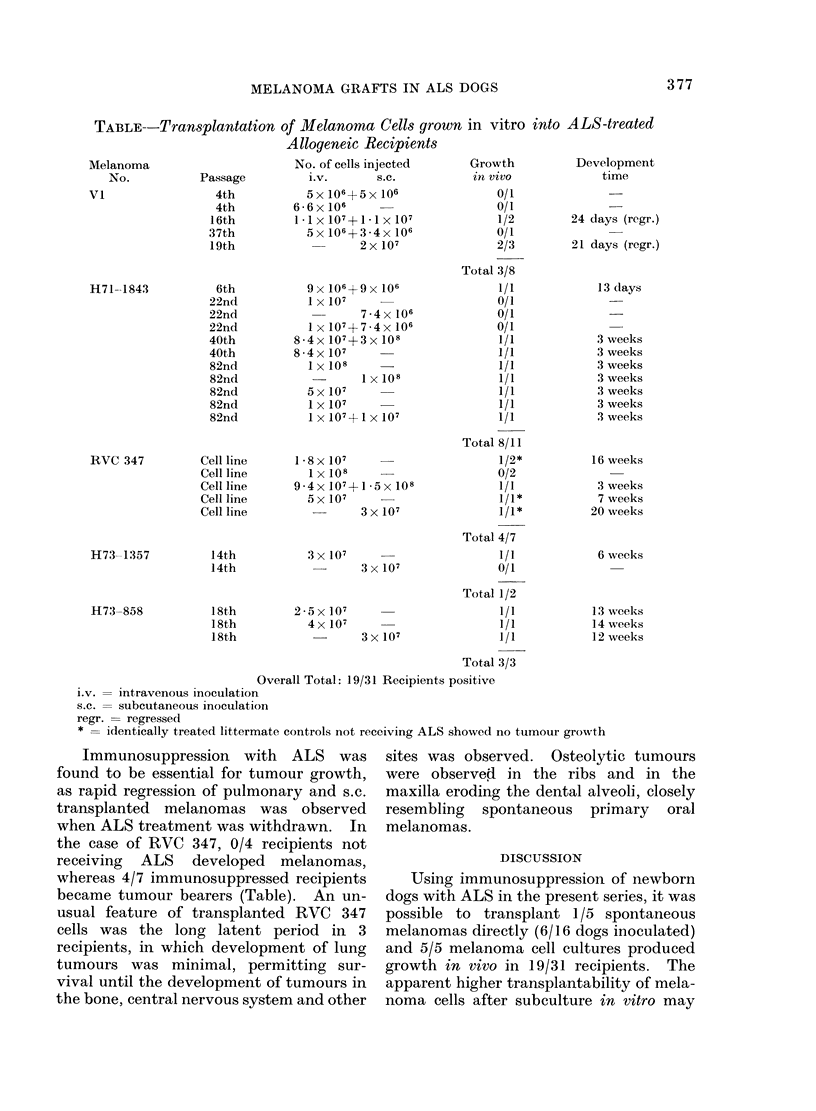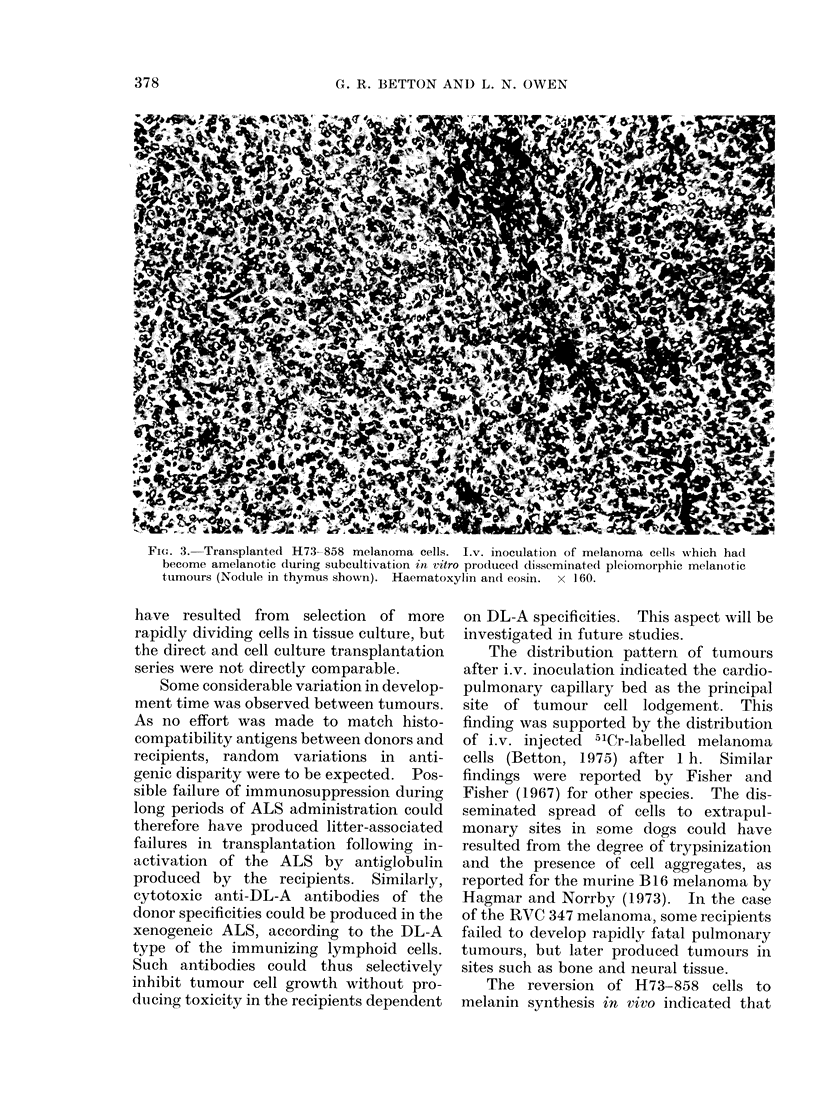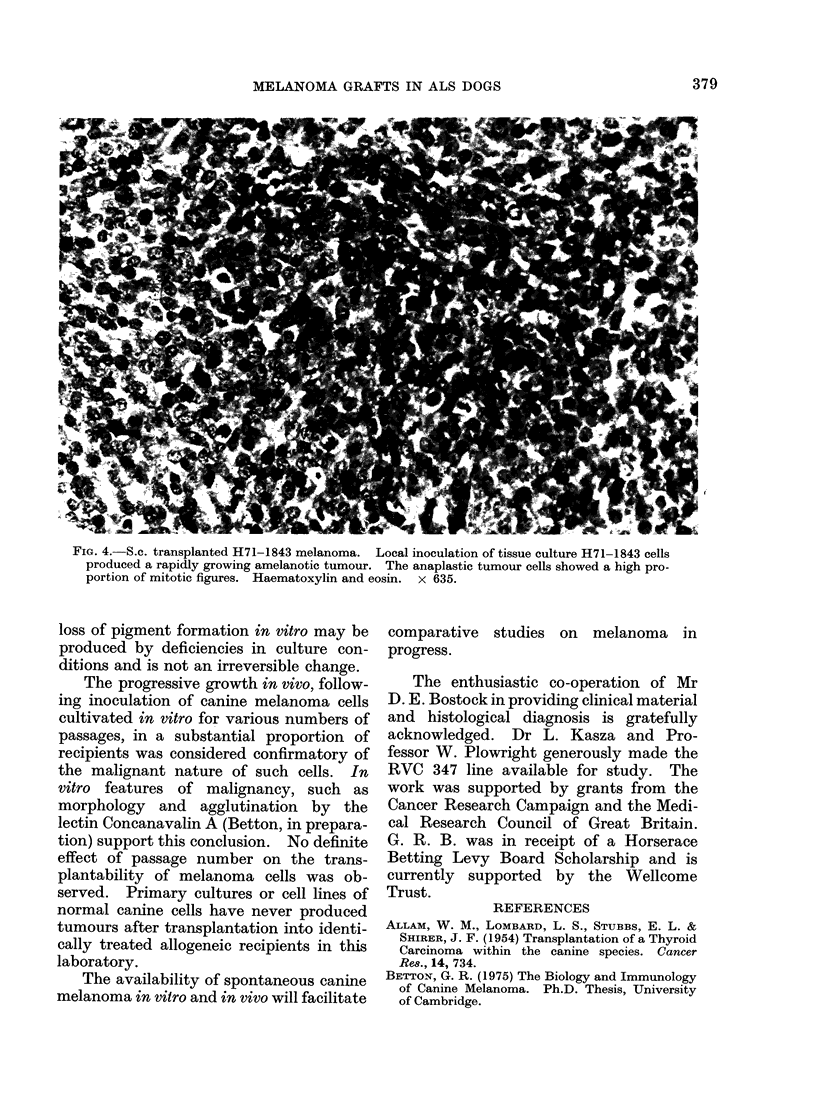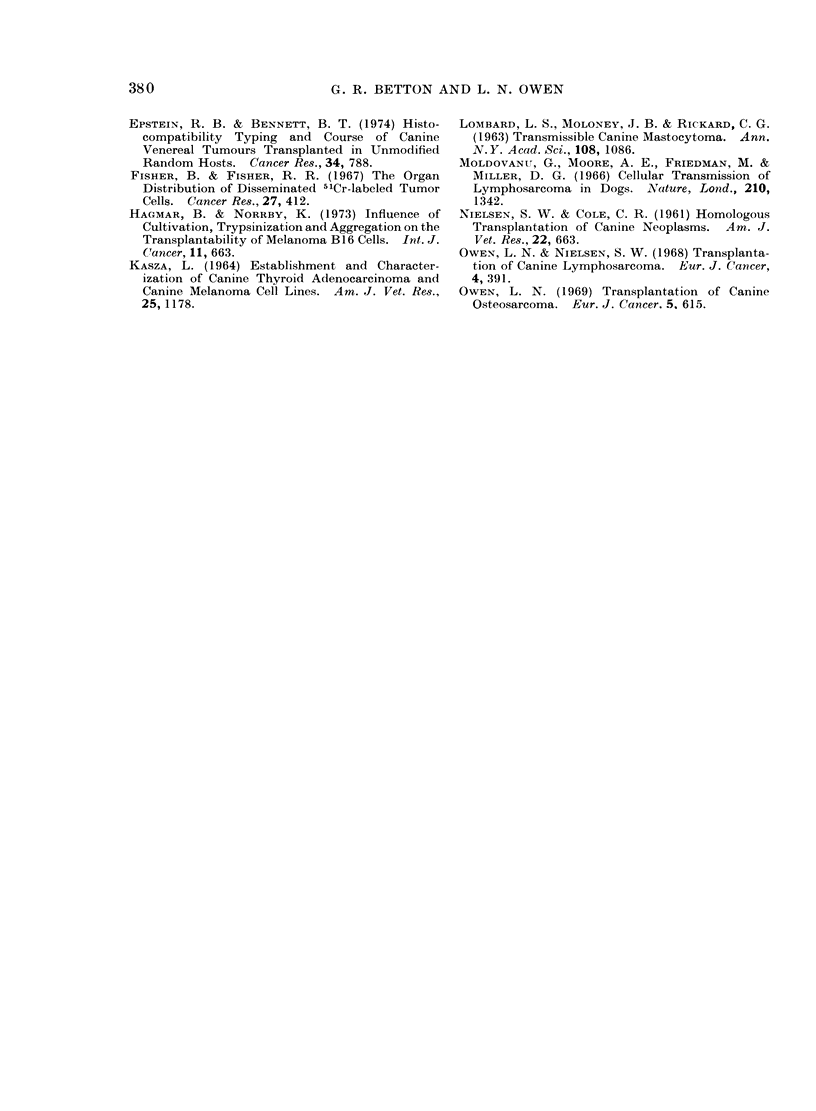# Allogeneic grafts of spontaneous canine melanomas and their cell culture strains in neonatal immunosuppressed dogs.

**DOI:** 10.1038/bjc.1976.180

**Published:** 1976-10

**Authors:** G. R. Betton, L. N. Owen

## Abstract

**Images:**


*Br. J. Cancer* (1976) **34**, 374

### ALLOGENEIC GRAFTS OF SPONTANEOUS CANINE MELANOMAS AND THEIR CELL CULTURE STRAINS IN NEONATAL IMMUNOSUPPRESSED DOGS

G. R. BETTON AND L. N. OWEN

*From the Department of Clinical Veterinary Medicine, University of Cambridge, England*

Received 5 April 1976  Accepted 3 June 1976

**Summary.—Canine melanoma has been transplanted to allogeneic neonatal recipients receiving continuous immunosuppression with anti-lymphocyte serum. One spontaneous melanoma was directly transplanted into 8 recipients, 6 of which developed tumours. 5/5 melanoma cell cultures were transplantable, with 19 tumour takes in 31 allogeneic recipients. Serial passage was performed in the case of two melanomas. Tumour development required continuous immunosuppression and the site was dependent upon the route of inoculation and other factors. Transplanted cell cultures were all amelanotic *in vitro* and *in vivo*, except in the case of one melanoma which reverted to a melanotic morphology after *in vivo* growth.**

CANINE spontaneous neoplasms have been infrequently transplanted, and success rates were low when irradiation or drug immunosuppression were employed (Allam *et al.*, 1954; Nielsen and Cole, 1961; Moldovanu *et al.*, 1966). Intrafoetal inoculation or immunosuppression with antilymphocyte serum (ALS) have yielded better results with canine osteosarcoma (Owen, 1969) and lymphosarcoma (Owen and Nielsen, 1968). Canine oral papilloma, mast cell leukaemia (Lombard, Moloney and Rickard, 1963) and the transmissible venereal tumour (Epstein and Bennett, 1974) are transplantable without immunosuppression. In many respects canine melanoma provides a valuable comparative model but has not previously been transplanted. Successful allogeneic transplantation into new-born ALS-treated dogs is now reported.

### MATERIALS AND METHODS

*Melanoma cells.*—Spontaneous cases of canine melanoma, involving the oropharynx or metastasizing to the regional lymph nodes, were excised aseptically under general anaesthesia. Cell suspensions for inoculation were prepared by finely cutting the melanoma tissue and pressing the cells through a 150-$\mu$m aperture sieve. Cells were either injected immediately after preparation or after cryopreservation at $-196°C$ in medium containing 10% DMSO.

Melanoma cells for *in vitro* cultivation were dissociated by sieving or by trypsinization. Cell cultures were grown as adherent monolayers in medium 199 or RPMI 1640 plus penicillin/streptomycin and 10% calf or foetal bovine serum and serially subcultured. Prior to inoculation, melanoma cells were detached with trypsin plus EDTA solution, washed and resuspended in Hanks' BSS.

*Recipients.*—Pregnant mongrel bitches were barrier-maintained and, following parturition, the newborn recipient dogs were placed on an immunosuppressive course of ALS treatment as previously reported (Owen, 1969) unless otherwise stated.

After estimating total and viable (trypan-blue-excluding) cell counts, melanoma cell suspensions were injected in Hanks' BSS *via* the jugular vein or s.c. on the lateral abdomen. Following inoculation, recipients were regularly checked for palpable s.c. tumour growth or symptoms of lethargy or dyspnoea indicative of systemic tumour growth. Thoracic radiography enabled early pulmonary tumour formation to be detected.

FIG. 1.—Transplantation and *in vivo* passage of a spontaneous canine melanoma. i.v. = intravenous inoculation. s.c. = subcutaneous inoculation.

### RESULTS

### *Transplantation directly from spontaneous melanomas*

In a series of 16 dogs inoculated with viable cells from 5 canine melanomas, tumour growth was observed in 6 animals. All successful transplants were derived from one donor (VI) either as fresh or cryopreserved cell suspensions (Fig.1). I.v. injection produced miliary lung tumours and myocardial infiltration along the coronary vessels. Pulmonary tumours exhibited various degrees of pigmentation. Some dogs also developed melanomas in the pancreas, mesentery, pleura and lymph nodes. S.c. inoculation produced large melanotic tumours which were locally invasive and, in one recipient, present in distant sites. Development time for detectable growth ranged from 3–13 weeks, except for one dog which remained clinically normal until euthanasia at 7 months of age, when pulmonary melanoma deposits were found. All tumours were confirmed histologically as melanomas. Subsequently, three serial *in vivo* passages were obtained with inoculation by the i.v. and s.c. routes (Fig. 1). All the remaining melanomas failed to grow after i.v. or s.c. inoculation of $10^8$ tumour cells into ALS-treated recipients.

FIG. 2.—Amelanotic melanoma cells (H71–1843) *in vitro*. Cells show a bipolar or multipolar morphology with the presence of some multinucleate and giant cells. Giemsa stain. × 320.

*Transplantation of canine melanoma cells grown* in vitro

Melanoma cell cultures were established from spontaneous amelanotic (H71–1843, H73–1357) and melanotic melanomas (V1, H73–858). Following continuous subcultivation *in vitro*, established cultures became amelanotic (Fig. 2) but retained other features of malignant melanoma cells (Betton, 1975). The RVC 347 melanoma cell line (Kasza, 1964) was also generously provided for study.

The results of inoculation of 5 canine melanoma cultures into immunosuppressed recipients are summarized in the Table. All the melanoma cultures tested showed *in vivo* growth in a proportion of recipients, although V1 tumours showed regression after the initial 3 week growth phase.

I.v. inoculation produced tumour growth in the lungs and frequently the myocardium and in some cases in the pleura, peritoneum, bone, pancreas, thymus (Fig.3), pelvic and perirenal lymph nodes and the central nervous system. Miliary s.c. deposits of melanoma have also been observed. Histopathological examination revealed the presence of anaplastic melanoma cells with frequent mitotic figures and devoid of melanin except in the case of H73–858 cells. Although the latter cells had become amelanotic during growth *in vitro*, the transplanted tumours were all heavily pigmented *in vivo* in s.c. and disseminated visceral sites.

S.c. tumour inoculation produced rapidly-growing localized tumours (Fig. 4), often locally invasive and sometimes ulcerating. Distant tumours were not observed, except in the case of H73–858 cells, when some lung tumours also developed. In the case of H71–1843, melanoma cells from transplanted tumour tissue were maintained in tissue culture for two passages and were subsequently capable of further growth *in vivo* when re-inoculated.

TABLE—*Transplantation of Melanoma Cells grown* in vitro *into ALS-treated Allogeneic Recipients*

| Melanoma No. | Passage | No. of cells injected i.v. | s.c. | Growth *in vivo* | Development time |
|---|---|---|---|---|---|
| V1 | 4th | $5\times10^6$ | $+5\times10^6$ | 0/1 | — |
| | 4th | $6\cdot6\times10^6$ | — | 0/1 | — |
| | 16th | $1\cdot1\times10^7$ | $+1\cdot1\times10^7$ | 1/2 | 24 days (regr.) |
| | 37th | $5\times10^6$ | $+3\cdot4\times10^6$ | 0/1 | — |
| | 19th | — | $2\times10^7$ | 2/3 | 21 days (regr.) |
| | | | | Total 3/8 | |
| H71–1843 | 6th | $9\times10^6$ | $+9\times10^6$ | 1/1 | 13 days |
| | 22nd | $1\times10^7$ | — | 0/1 | — |
| | 22nd | — | $7\cdot4\times10^6$ | 0/1 | — |
| | 22nd | $1\times10^7$ | $+7\cdot4\times10^6$ | 0/1 | — |
| | 40th | $8\cdot4\times10^7$ | $+3\times10^8$ | 1/1 | 3 weeks |
| | 40th | $8\cdot4\times10^7$ | — | 1/1 | 3 weeks |
| | 82nd | $1\times10^8$ | — | 1/1 | 3 weeks |
| | 82nd | — | $1\times10^8$ | 1/1 | 3 weeks |
| | 82nd | $5\times10^7$ | — | 1/1 | 3 weeks |
| | 82nd | $1\times10^7$ | — | 1/1 | 3 weeks |
| | 82nd | $1\times10^7$ | $+1\times10^7$ | 1/1 | 3 weeks |
| | | | | Total 8/11 | |
| RVC 347 | Cell line | $1\cdot8\times10^7$ | — | 1/2* | 16 weeks |
| | Cell line | $1\times10^8$ | — | 0/2 | — |
| | Cell line | $9\cdot4\times10^7$ | $+1\cdot5\times10^8$ | 1/1 | 3 weeks |
| | Cell line | $5\times10^7$ | — | 1/1* | 7 weeks |
| | Cell line | — | $3\times10^7$ | 1/1* | 20 weeks |
| | | | | Total 4/7 | |
| H73–1357 | 14th | $3\times10^7$ | — | 1/1 | 6 weeks |
| | 14th | — | $3\times10^7$ | 0/1 | — |
| | | | | Total 1/2 | |
| H73–858 | 18th | $2\cdot5\times10^7$ | — | 1/1 | 13 weeks |
| | 18th | $4\times10^7$ | — | 1/1 | 14 weeks |
| | 18th | — | $3\times10^7$ | 1/1 | 12 weeks |
| | | | | Total 3/3 | |

Overall Total: 19/31 Recipients positive i.v. = intravenous inoculation
s.c. = subcutaneous inoculation
regr. = regressed
* = identically treated littermate controls not receiving ALS showed no tumour growth

Immunosuppression with ALS was found to be essential for tumour growth, as rapid regression of pulmonary and s.c. transplanted melanomas was observed when ALS treatment was withdrawn. In the case of RVC 347, 0/4 recipients not receiving ALS developed melanomas, whereas 4/7 immunosuppressed recipients became tumour bearers (Table). An unusual feature of transplanted RVC 347 cells was the long latent period in 3 recipients, in which development of lung tumours was minimal, permitting survival until the development of tumours in the bone, central nervous system and other sites was observed. Osteolytic tumours were observed in the ribs and in the maxilla eroding the dental alveoli, closely resembling spontaneous primary oral melanomas.

### DISCUSSION

Using immunosuppression of newborn dogs with ALS in the present series, it was possible to transplant 1/5 spontaneous melanomas directly (6/16 dogs inoculated) and 5/5 melanoma cell cultures produced growth *in vivo* in 19/31 recipients. The apparent higher transplantability of melanoma cells after subculture *in vitro* may

FIG. 3.—Transplanted H73–858 melanoma cells. I.v. inoculation of melanoma cells which had become amelanotic during subcultivation *in vitro* produced disseminated pleiomorphic melanotic tumours (Nodule in thymus shown). Haematoxylin and eosin. × 160.

have resulted from selection of more rapidly dividing cells in tissue culture, but the direct and cell culture transplantation series were not directly comparable.

Some considerable variation in development time was observed between tumours. As no effort was made to match histocompatibility antigens between donors and recipients, random variations in antigenic disparity were to be expected. Possible failure of immunosuppression during long periods of ALS administration could therefore have produced litter-associated failures in transplantation following inactivation of the ALS by antiglobulin produced by the recipients. Similarly, cytotoxic anti-DL-A antibodies of the donor specificities could be produced in the xenogeneic ALS, according to the DL-A type of the immunizing lymphoid cells. Such antibodies could thus selectively inhibit tumour cell growth without producing toxicity in the recipients dependent on DL-A specificities. This aspect will be investigated in future studies.

The distribution pattern of tumours after i.v. inoculation indicated the cardiopulmonary capillary bed as the principal site of tumour cell lodgement. This finding was supported by the distribution of i.v. injected $^{51}$Cr-labelled melanoma cells (Betton, 1975) after 1 h. Similar findings were reported by Fisher and Fisher (1967) for other species. The disseminated spread of cells to extrapulmonary sites in some dogs could have resulted from the degree of trypsinization and the presence of cell aggregates, as reported for the murine B16 melanoma by Hagmar and Norrby (1973). In the case of the RVC 347 melanoma, some recipients failed to develop rapidly fatal pulmonary tumours, but later produced tumours in sites such as bone and neural tissue.

The reversion of H73–858 cells to melanin synthesis *in vivo* indicated that

FIG. 4.—S.c. transplanted H71–1843 melanoma. Local inoculation of tissue culture H71–1843 cells produced a rapidly growing amelanotic tumour. The anaplastic tumour cells showed a high proportion of mitotic figures. Haematoxylin and eosin. × 635.

loss of pigment formation *in vitro* may be produced by deficiencies in culture conditions and is not an irreversible change.

The progressive growth *in vivo*, following inoculation of canine melanoma cells cultivated *in vitro* for various numbers of passages, in a substantial proportion of recipients was considered confirmatory of the malignant nature of such cells. *In vitro* features of malignancy, such as morphology and agglutination by the lectin Concanavalin A (Betton, in preparation) support this conclusion. No definite effect of passage number on the transplantability of melanoma cells was observed. Primary cultures or cell lines of normal canine cells have never produced tumours after transplantation into identically treated allogeneic recipients in this laboratory.

The availability of spontaneous canine melanoma *in vitro* and *in vivo* will facilitate comparative studies on melanoma in progress.

The enthusiastic co-operation of Mr D. E. Bostock in providing clinical material and histological diagnosis is gratefully acknowledged. Dr L. Kasza and Professor W. Plowright generously made the RVC 347 line available for study. The work was supported by grants from the Cancer Research Campaign and the Medical Research Council of Great Britain. G. R. B. was in receipt of a Horserace Betting Levy Board Scholarship and is currently supported by the Wellcome Trust.

### REFERENCES

ALLAM, W. M., LOMBARD, L. S., STUBBS, E. L. & SHIRER, J. F. (1954) Transplantation of a Thyroid Carcinoma within the canine species. *Cancer Res.*, **14**, 734.

BETTON, G. R. (1975) The Biology and Immunology of Canine Melanoma. Ph.D. Thesis, University of Cambridge.

EPSTEIN, R. B. & BENNETT, B. T. (1974) Histocompatibility Typing and Course of Canine Venereal Tumours Transplanted in Unmodified Random Hosts. *Cancer Res.*, **34**, 788.

FISHER, B. & FISHER, R. R. (1967) The Organ Distribution of Disseminated $^{51}$Cr-labeled Tumor Cells. *Cancer Res.*, **27**, 412.

HAGMAR, B. & NORRBY, K. (1973) Influence of Cultivation, Trypsinization and Aggregation on the Transplantability of Melanoma B16 Cells. *Int. J. Cancer*, **11**, 663.

KASZA, L. (1964) Establishment and Characterization of Canine Thyroid Adenocarcinoma and Canine Melanoma Cell Lines. *Am. J. Vet. Res.*, **25**, 1178.

LOMBARD, L. S., MOLONEY, J. B. & RICKARD, C. G. (1963) Transmissible Canine Mastocytoma. *Ann. N.Y. Acad. Sci.*, **108**, 1086.

MOLDOVANU, G., MOORE, A. E., FRIEDMAN, M. & MILLER, D. G. (1966) Cellular Transmission of Lymphosarcoma in Dogs. *Nature, Lond.*, **210**, 1342.

NIELSEN, S. W. & COLE, C. R. (1961) Homologous Transplantation of Canine Neoplasms. *Am. J. Vet. Res.*, **22**, 663.

OWEN, L. N. & NIELSEN, S. W. (1968) Transplantation of Canine Lymphosarcoma. *Eur. J. Cancer*, **4**, 391.

OWEN, L. N. (1969) Transplantation of Canine Osteosarcoma. *Eur. J. Cancer*, **5**, 615.